# Supplementary material for: Compensatory metabolic networks in pancreatic cancers upon perturbation of glutamine metabolism
Source: Nat Commun. 2017 Jul 3;8:15965. doi: 10.1038/ncomms15965 (PMC5500878; doi:10.1038/ncomms15965)
Supplement: Supplementary Information [file ncomms15965-s1.pdf]

Type of file: PDF
Size of file: 0 KB
Title of file for HTML: Supplementary Information
Description: Supplementary Figures

Type of file: XLSX
Size of file: 0 KB
Title of file for HTML: Supplementary Data 1
Description: Metabolomics data for CB-839-treated MPDAC-4 (in vitro and in vivo (flank) tumors) and PaTu-8988T cells as represented in Fig. 1, 4 and Supplementary Fig. 3. Below are representative MPDAC-4 and PaTu-8988T datasets (of 3 experiments for cell culture data and the one in vivo experiment) with log2 changes (CB-839/control).

Type of file: XLSX
Size of file: 0 KB
Title of file for HTML: Supplementary Data 2
Description: Metaboanalyst metabolite set enrichment analysis (MSEA) from Fig. 4d (MPDAC-4-in vitro, upregulated), Fig. 4g (MPDAC-4-in vivo-upregulated), Supplementary Fig. 4d (MPDAC-4-in vitro-downregulated), and Supplementary Fig. 5a (PatTu-8988T-in vitro upregulated).

Type of file: XLSX
Size of file: 0 KB
Title of file for HTML: Supplementary Data 3
Description: Metabolomics data for CB-839-treated MPDAC-4 U13C-Gln tracing experiment, related to Supplementary Fig. 3e,f, Pooled data, fractional data, and raw peak data presented. NF = not found.

Type of file: XLSX
Size of file: 0 KB
Title of file for HTML: Supplementary Data 4
Description: TMT proteomic raw data and comparisons between DMSO- and CB-839-treated MPDAC-4 cells treated for 24 or 72 hours. Comparisons were performed with a t-test with Bonferroni correction for the number of peptides with more than 1 detected peptide. False discovery rates (FDRs) were calculated using the Benjamini-Hochberg procedure. Columns include: Uniprot protein identification number (proteinID), gene symbol (Gene Symbol), protein description/name (Description), number of peptides quantified per protein (peptides), the normalized summed signal-to-noise (sn) for each of the 9 channels (126 to 130c), the mean sum signal-to-noise of TMT relative abundance values for each treatment, the fold-change comparisons of the CB-839 treated to DMSO treated samples, ANOVA p-value across the 3 treatments, Bonferroni-adjusted p values, and Benjamini-Hochberg calculated FDRs. From left to right, experiment 1, 2, and 3.

Type of file: XLSX
Size of file: 0 KB
Title of file for HTML: Supplementary Data 5
Description: Gene set enrichment analysis (GSEA) of CB-839-72h vs. DMSO quantitative proteomic comparison (Experiment 1). Gene sets with a false discovery rate <0.01 and coefficient score >0.25 were entered into enrichment map analysis (Fig. 5d). From left to right as labeled, genesets associated with upregulated proteins, genesets associated with downregulated proteins, genesets represented within each 'cloud' in Fig. 5d.

Type of file: XLSX
Size of file: 0 KB
Title of file for HTML: Supplementary Data 6
Description: TMT proteomic raw data and comparisons between DMSO- and CB-839-treated PaTu-8988T cells treated for 72 hours or at least 15 days (Resistant (R)). Comparisons were performed with a t-test with Bonferroni correction for the number of peptides with more than 1 detected peptide. False discovery rates (FDRs) were calculated using the Benjamini-Hochberg procedure. Columns include: Uniprot protein identification number (proteinID), gene symbol (Gene Symbol), protein description/name (Description), number of peptides quantified per protein (peptides), the normalized summed signal-to-noise (sn) for each of the 9 channels (126 to 130c), the mean sum signal-to-noise of TMT relative abundance values for each treatment, the fold-change comparisons of the CB-839 treated to DMSO treated samples, ANOVA p-value across the 3 treatments, Bonferroni-adjusted p values, and Benjamini-Hochberg calculated FDRs.

Type of file: XLSX
Size of file: 0 KB
Title of file for HTML: Supplementary Data 7
Description: Quantitative proteomics of MDPAC-4 and PaTu-8988T cell lines after CB-839 treatment reveal alterations in glutamate-producing enzymes (associated with Supplementary Fig. 4d-f), oxidative stress response related proteins (associated with Supplementary Fig. 6a), and fatty acid metabolic processes (associated with Supplementary Fig. 6b). Data are derived from experimental data in Supplementary Data 4, 6, and 9 and represented here.

Type of file: XLSX
Size of file: 0 KB
Title of file for HTML: Supplementary Data 8
Description: Metaboanalyst integrated pathway analysis of MPDAC-4 CB-839 72 h quantitative proteomics (Experiment 1, from Supplementary Data 4) top 5% significantly upregulated and downregulated proteins combined with MPDAC-4 CB-839 4 h metabolite dataset from Fig. 4b (from Supplementary Data 1).

Type of file: XLSX
Size of file: 0 KB
Title of file for HTML: Supplementary Data 9
Description: TMT proteomic raw data and comparisons between DMSO- and CB-839-treated MPDAC-4 cells treated for 72 hours or at least 15 days (Resistant (R)). Comparisons were performed with a t-test with Bonferroni correction for the number of peptides with more than 1 detected peptide. False discovery rates (FDRs) were calculated using the Benjamini-Hochberg procedure. Columns include: Uniprot protein identification number (proteinID), gene symbol (Gene Symbol), protein description/name (Description), number of peptides quantified per protein (peptides), the normalized summed signal-to-noise (sn) for each of the 9 channels (126 to 130c), the mean sum signal-to-noise of TMT relative abundance values for each treatment, the fold-change comparisons of the CB-839 treated to DMSO treated samples, ANOVA p-value across the 3 treatments, Bonferroni-adjusted p values, and Benjamini-Hochberg calculated FDRs.

Type of file: XLSX
Size of file: 0 KB
Title of file for HTML: Supplementary Data 10
Description: Gene set enrichment analysis (GSEA) of CB-839-72h vs. CB-839-R (Resistant) vs. DMSO quantitative proteomic comparison (dataset in Supplementary Data 9). For the CB-839-R vs. DMSO comparison, gene sets with a false discovery rate <0.05 and coefficient score >0.1 were entered into

enrichment map analysis (Fig. 7b, see top, left to right). For the CB-839-R vs. CB-839-72h comparison, gene sets with a false discovery rate <0.01 and coefficient score >0.25 were entered into enrichment map analysis (Fig. 7c, see bottom, left to right).

Type of file: XLSX
Size of file: 0 KB
Title of file for HTML: Supplementary Data 11
Description: Results of the Connectivity Map 2.0 (CMAP) analysis. CMAP analysis for proteins that showed up- or downregulation in the three experiments for the MPDAC-4 DMSO vs. CB-839-24h vs. CB-839-72h treatment (from Supplementary Data 4, presented in Fig. 7e, f). CMAP analysis for the MPDAC-4 DMSO vs. CB-839-72h vs. CB-839-R (from Supplementary Data 9, presented in Fig. 7g,h). Data are presented as mean connectivity score between the lists of proteins with altered expression due to CB-839 treatment and gene expression changes elicited by drugs listed in a given row. FDR - false discovery rate. Significant (FDR<0.05) correlations were marked in red. See Fig. 7d and methods for workflow.

Type of file: XLSX
Size of file: 0 KB
Title of file for HTML: Supplementary Data 12
Description: Compilation of statistical results from Fig. 1-4, Fig. 6-7, and Supplementary Fig. 1-6.

Type of file: pdf
Size of file: 0 KB
Title of file for HTML: Peer Review File
Description:

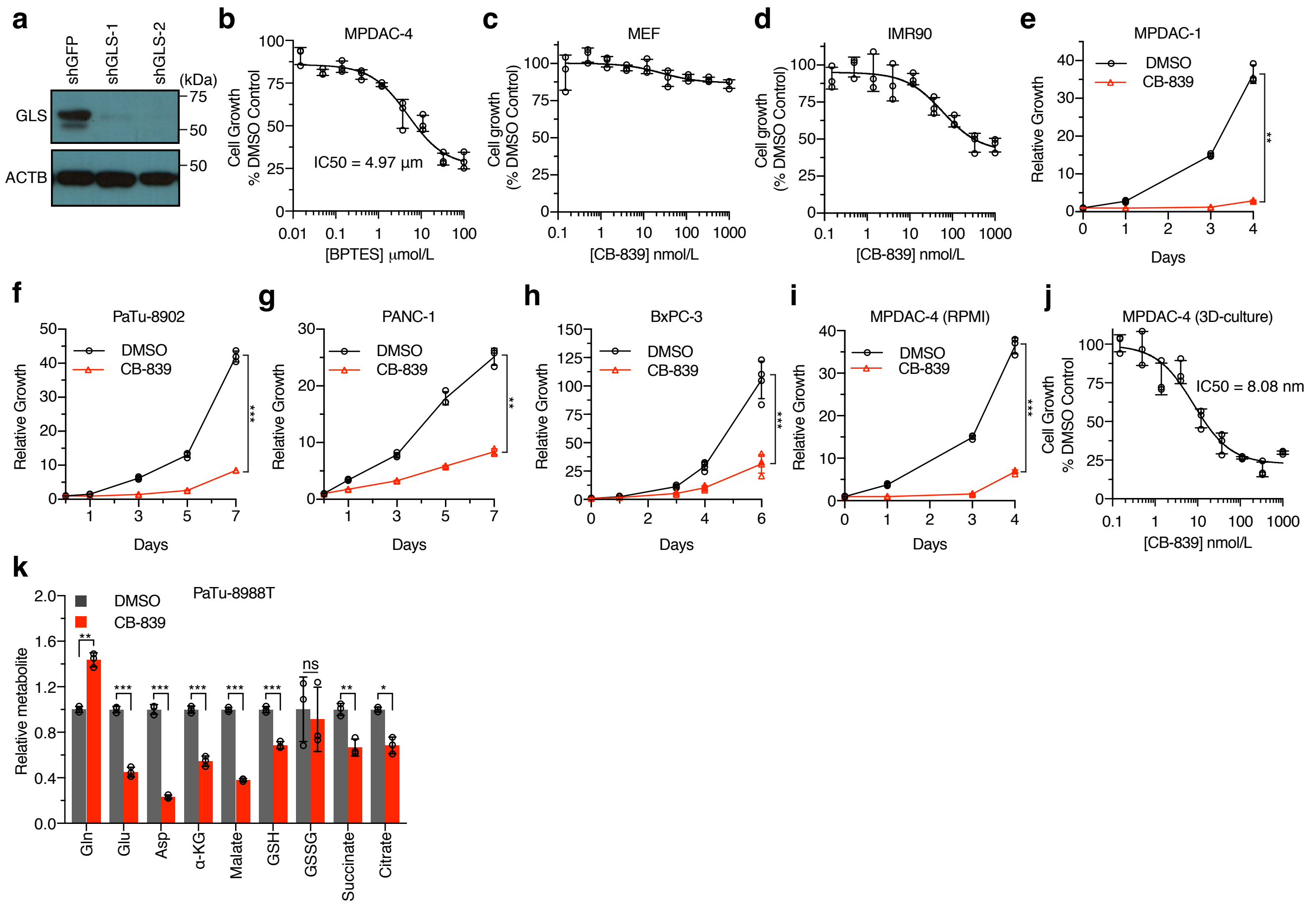


**Supplementary Figure 1 PDAC cell lines are sensitive to GLS pharmacologic inhibition with CB-839**

(**a**) GLS western blot analysis of extracts from PaTu-8988T cells as in Fig. 1a expressing a control shRNA or shRNAs to GLS (#1 and #2). (**b**) Cell proliferation dose-response curve for MPDAC-4 treated with BPTES for 72 h. Error bars depict ± s.d. of 3 independent wells from a representative experiment (of 3 experiments). (**c**, **d**) Cell proliferation dose-response curves for mouse embryonic fibroblasts (MEF) (c) and IMR90 (d) treated with CB-839 for 72 h. Error bars depict ± s.d. of 3 independent wells from a representative experiment (of 3 experiments). (**e-i**) Relative proliferation of PDAC cell lines all treated with CB-839 or DMSO, (**e**) MPDAC-1 cell line derived from a LSL-KrasG12D; p53 L/+, Pdx1-Cre tumor-bearing mouse, (**f**) PaTu-8902, (**g**) PANC-1, (**h**) BxPC-3 (KRas wild-type), (**i**) MPDAC-4 cultured in RPMI media. Data are plotted as mean relative cell proliferation ± s.d. of 3 independent wells (4 wells for h) from representative experiments (of 3 experiments). Significance determined by t-Test in panels comparing last time points. * P<0.05, ** P<0.01, *** P<0.001, ns: non-significant, P>0.05. (**j**) Cell proliferation dose-response curves for MPDAC-4 cells grown in 3D-culture with matrigel treated with CB-839 for 72 h, IC50=8.08 nm. Error bars depict ± s.d. of 3 independent wells from a representative experiment (of 3 experiments). (**k**) Relative metabolite abundance in PaTu-8988T cells following 6 h CB-839 treatment. Data are presented as mean total metabolite pools ± s.d. of 3 independent wells from a representative experiment (of 3 experiments). Significance determined by t-Test * P<0.05, ** P<0.01, *** P<0.001, ns: non-significant, P>0.05.

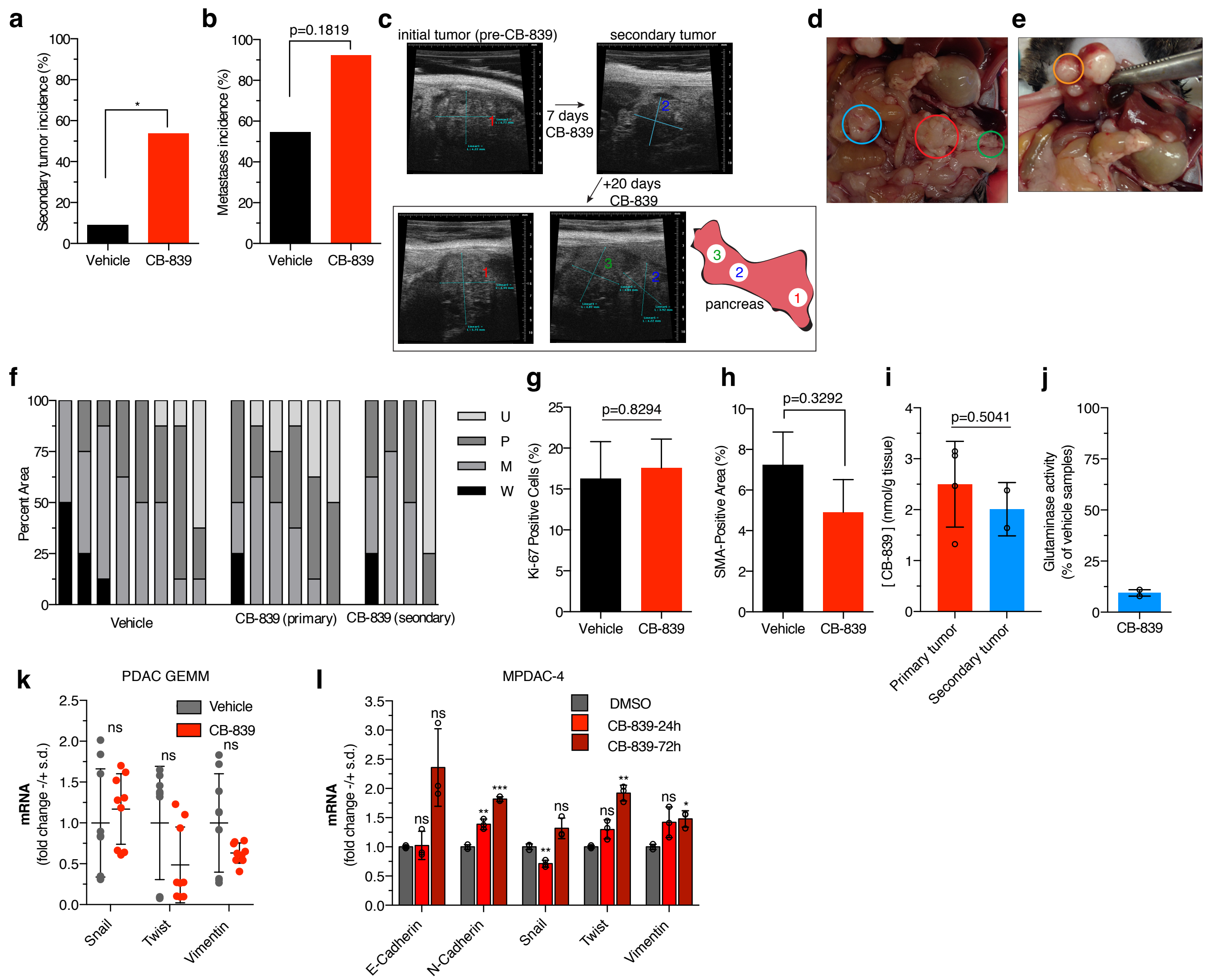

**Supplementary Figure 2 PDAC mice treated with CB-839 develop second primary PDAC tumors**

(**a**) Secondary tumor incidence from mice from experiment in Fig. 2d, vehicle, n=11, CB-839, n=13, P=0.0335. (**b**) Incidence of macrometastases from mice in Fig. 2d, P=0.1819. For (a,b) comparisons using a two-tailed Fisher's exact test. (**c**) High-resolution ultrasound of a mouse treated with CB-839 that developed multiple secondary tumors after initiation of CB-839 (primary tumor numbered (1), secondary tumors numbered (2) and (3) respectively. (**d**) Autopsy of a mouse treated with CB-839 for 18 days on the trial before endpoint was reached. A red circle demarcates the initial pancreatic tumor. Blue and green circles demarcate secondary pancreatic tumors. (**e**) Liver macrometastases identified at autopsy. (**f**) No difference in differentiation between tumors of mice with vehicle versus CB-839 including primary tumors and secondary tumors. Quantification of differentiation state, the fraction of each tumor that was observed to be well-differentiated (W), moderately differentiated (M), poorly differentiated (P), or undifferentiated (U) was scored in a blinded manner, and compared between the cohorts. (**g**) No difference in proliferative index in CB-839 treated tumors. Quantification of Ki-67 positive cells as a percentage of all nucleated tumor cells (vehicle, n=5, CB-839, n=5 bars represent mean ± s.e.m.), P=0.8294 (t-Test). (**h**) No difference in SMA-positive tumor stroma area in CB-839 treated tumors. Quantification of SMA positive area as a percentage of field area (vehicle, n=5, CB-839, n=5 bars represent mean ± s.e.m.), P=0.3292 (t-Test). (**i**) CB-839 levels in primary and secondary tumor samples of mice treated with CB-839, primary tumor, n=4, secondary tumor, n=2, data represented as mean ± s.d., P=0.5041 (t-Test). (**j**) Glutaminase activity measured in tumor lysates from secondary tumors (n=2). The percent inhibition by CB-839 is relative to vehicle data from Fig. 2b. data represented as mean ± s.d, P=0.0614 (t-Test) in comparison to vehicle from Fig. 2b. (**k**) Markers of epithelial-mesenchymal transition (EMT) assessed by RT-qPCR from tumors harvested from LSL-KrasG12D; p53 L/+, Pdx1-Cre mice treated with vehicle (n=3) or CB-839 (n=3) from Fig. 2d. Expression levels are normalized to 18S ribosomal RNA and presented as mean ± s.d. of 3 tumors tested in triplicate. (**l**) Markers of EMT assessed by RT-qPCR in MPDAC-4 cells treated as indicated (1 $\mu$m CB-839). Expression levels are normalized to 18S ribosomal RNA and presented as mean ± s.d. of 3 independent wells from a representative experiment (of 3 experiments). Significance determined by t-Test for (k,l), * P<0.05, ** P<0.01, *** P<0.001, ns: non-significant, P>0.05.

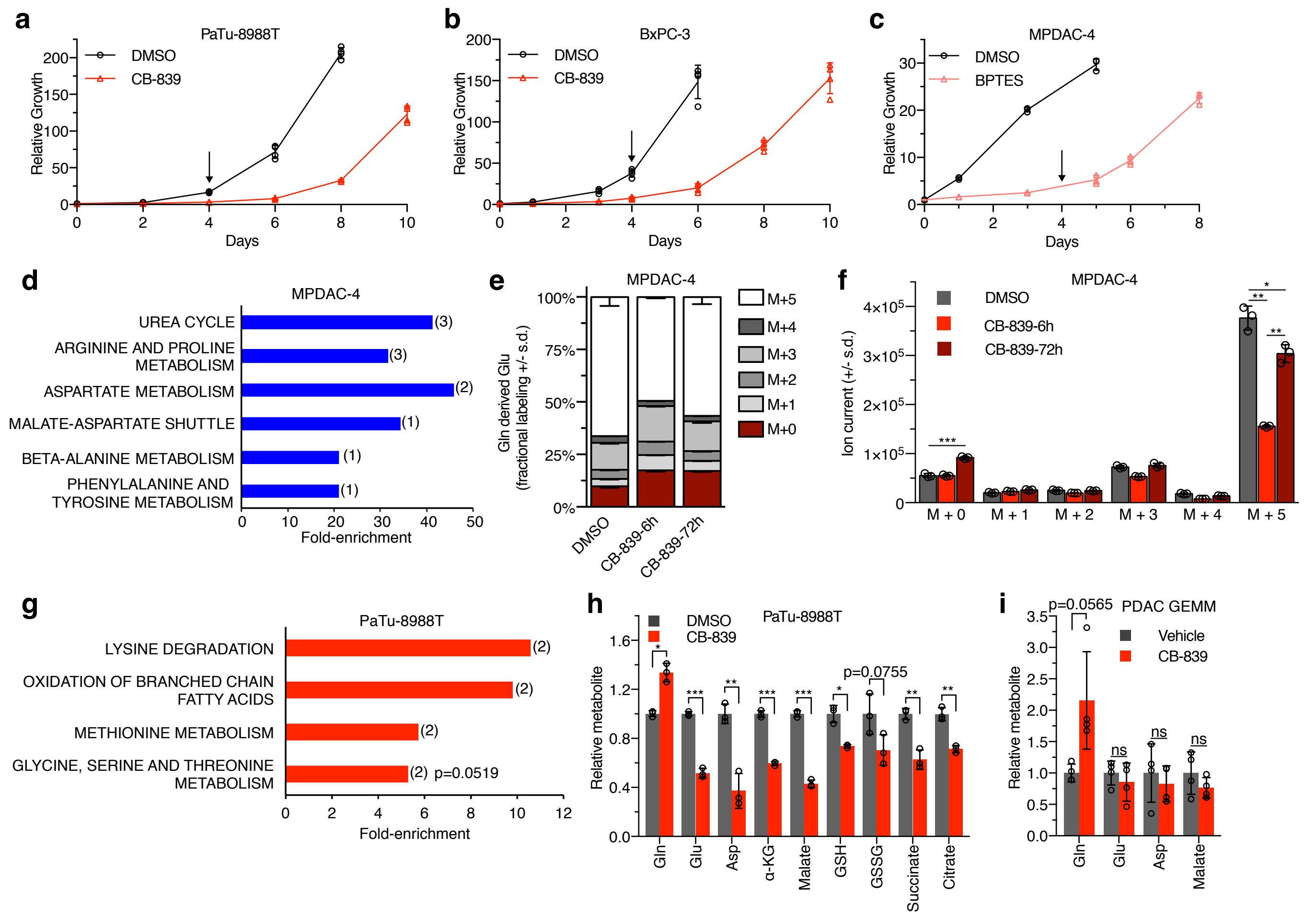


**Supplementary Figure 3 Metabolic profiling of PDAC cells after GLSi identifies upregulated pathways**
(**a**, **b**) Relative proliferation of (a) PaTu-8988T or (b) BxPC-3 cell line treated long-term with CB-839 or DMSO. Arrow represents time point when treatment was refreshed (similar in c). (**c**) Relative proliferation of MPDAC-4 cell line treated long-term with BPTES or DMSO. Error bars in (a-c) depict ± s.d. of 3 independent wells from a representative experiment (of 3 experiments). (**d**) Metabolite set enrichment analysis (MSEA) of significantly downregulated metabolites from MPDAC-4 72 h CB-839 metabolomics experiment as in Fig. 4b. Statistically significant terms are graphed according to fold-enrichment (number of metabolites represented in term in parentheses). (**e**,**f**) Metabolic tracing studies using uniformly 13C-labeled Gln. The results for 13C-labeled Glu metabolites are plotted for cells treated with DMSO versus CB-839-6h, CB-839-72h. (**e**) fractional data presented ± s.d., (**f**) mean ion current ± s.d. of triplicate wells from a representative of at least 2 experiments, DMSO vs. CB-839-72h, M+0: P=0.00056, DMSO vs. CB-839-6h, M+5: P=0.00389, DMSO vs. CB-839-72h, M+5: P=0.01727, CB-839-6h vs. CB-839-72h, M+5: P=0.00466. (**g**) MSEA of significantly upregulated metabolites from PaTu-8988T 72 h CB-839 metabolomics experiment (number of metabolites represented in term in parentheses). (**h**) Relative metabolite abundance in PaTu-8988T cells following 72 h CB-839 treatment. Data are presented as means of total metabolite pools ± s.d. of 3 independent wells from a representative experiment (of 3 experiments). (i) Relative metabolite abundance in tumor lysates of primary tumors from LSL-KrasG12D; p53 L/+, Pdx1-Cre mice bearing pancreatic tumors treated with vehicle or CB-839 for a minimum of two weeks (n=4 tumors per group), mice from clinical trial presented in Fig. 2d. Means of total metabolite pools ± s.d. presented. For (f, h, i) significance determined by t-Test, * P<0.05, ** P<0.01, *** P<0.001, ns: non-significant, P>0.05.

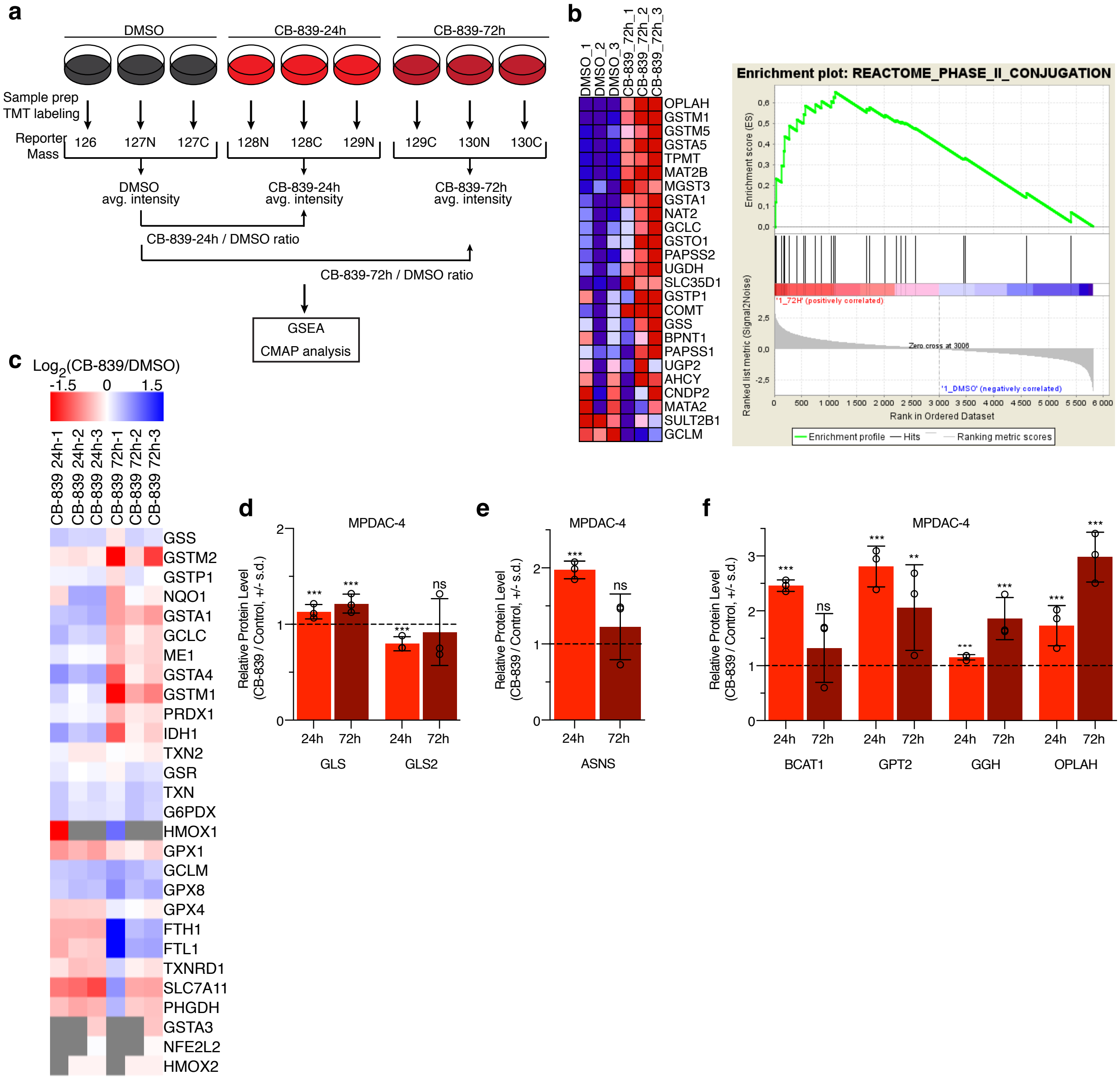


**Supplementary Figure 4 PDAC GLSi proteomic profiling identifies upregulated and downregulated pathways**
(**a**) Schematic of multiplexed TMT quantitative proteomics experiment. (**b**) GSEA heat map and enrichment analysis plot for Phase II Conjugation gene set (Reactome gene set involved in detoxification conjugation/transferase reactions) upregulated in CB-839-72h. (**c**) Heatmap of Nrf2 pathway proteins involved in oxidative stress response, data plotted from Supplementary Data 4, MPDAC-4 Experiments 1-3. Values presented are the mean of Log2(CB-839 treated sample / control) values from all three experiments. (**d**) GLS and GLS2 proteins are minimally changed in CB-839 treated cells. Data are derived from experimental data in Supplementary Data 4 (Experiments 1-3) and summarized in Supplementary Data 7, GLS-24h vs. DMSO, P=0.000286, GLS-72h vs. DMSO, P=3.63x10-5. (**e**) ASNS is upregulated approximately 2-fold in response to CB-839 at 24 h. Data are derived from experimental data in Supplementary Data 4 and summarized in Supplementary Data 7, ASNS-24h vs. DMSO, P=3.64x10-6, ASNS-72h vs. DMSO, P=0.4499. (**f**) Glutamine-independent glutamate producing enzymes are upregulated in response to CB-839 in MPDAC-4 cells. Data are derived from experimental data in Supplementary Data 4 and summarized in Supplementary Data 7, BCAT1-24h vs. DMSO, P= P=2.60x10-11, BCAT1-72h vs. DMSO, P=0.1220, GPT2-24h vs. DMSO, P=1.50x10-7, GPT2-72h vs. DMSO, P=0.001581, GGH-24h vs. DMSO, P=4.43x10-6, GGH-72h vs. DMSO P=5.07x10-5, OPLAH-24h vs. DMSO, P=0.000136, OPLAH-72h, P=2.22x10-6, for (d-f), mean fold-change ± s.d. is presented, P values are calculated using a t-Test comparing normalized values of CB-839 treated samples vs. DMSO from Experiments 1-3, * P<0.05, ** P<0.01, *** P<0.001, ns: non-significant, P>0.05.

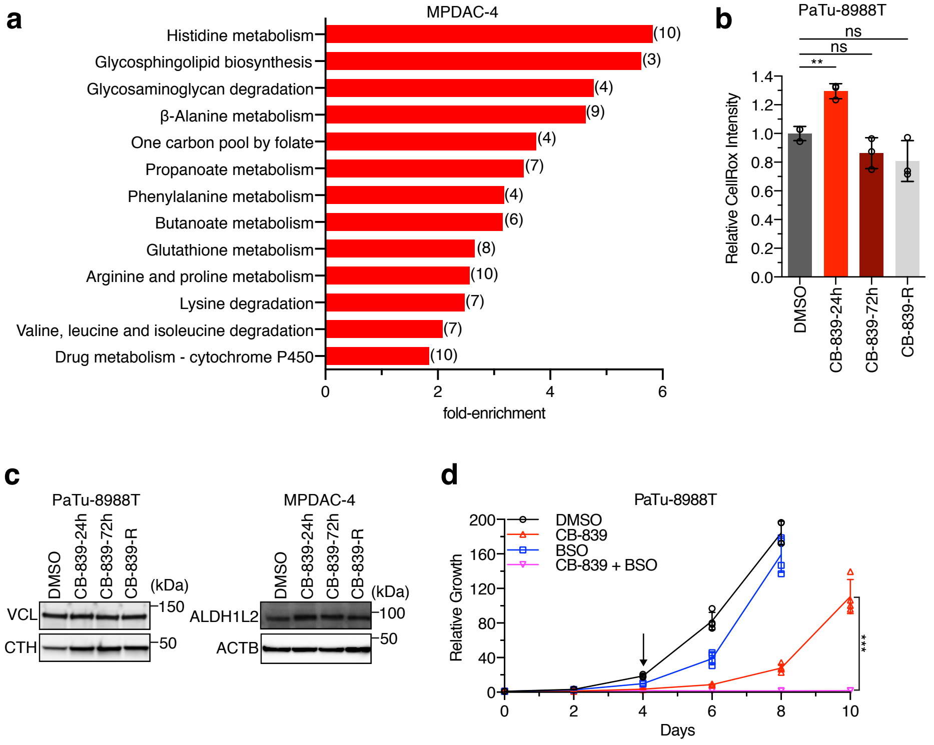


**Supplementary Figure 5 PDAC cell lines upregulate oxidative stress response pathways in response to GLSi**
(**a**) Metaboanalyst integrated pathway analysis of MPDAC-4 top 5% upregulated and down-regulated proteins combined with the up- and downregulated metabolites from the MPDAC-4 CB-839 4 h metabolite dataset from Fig. 4b (**b**) Relative CellRox intensity in PaTu-8988T cells following CB-839 treatment at the indicated times as measured by flow cytometry. Data are presented as mean ± s.d. of 3 independent wells from a representative experiment (of 3 experiments), CB-839-24h vs. DMSO, P=0.00208, CB-839-72h vs. DMSO, P=0.1454, CB-839-R vs. DMSO, P=0.1335. (**c**) Lysates from MPDAC-4 and PaTu-8988T cells treated with DMSO or CB-839 (1 µm) were analyzed using antibodies to ALDH1L2, CTH, ACTB (β-actin loading control for MPDAC-4), and VCL (Vinculin loading control for PaTu-8988T). (**d**) Relative proliferation of PaTu-8988T cell line treated with CB-839 or DMSO with or without BSO, CB-839 vs. CB-839 + BSO, Day 10, P=0.00160. Significance determined by t-Test for (b, d), ** P<0.01, ns: non-significant, P>0.05.

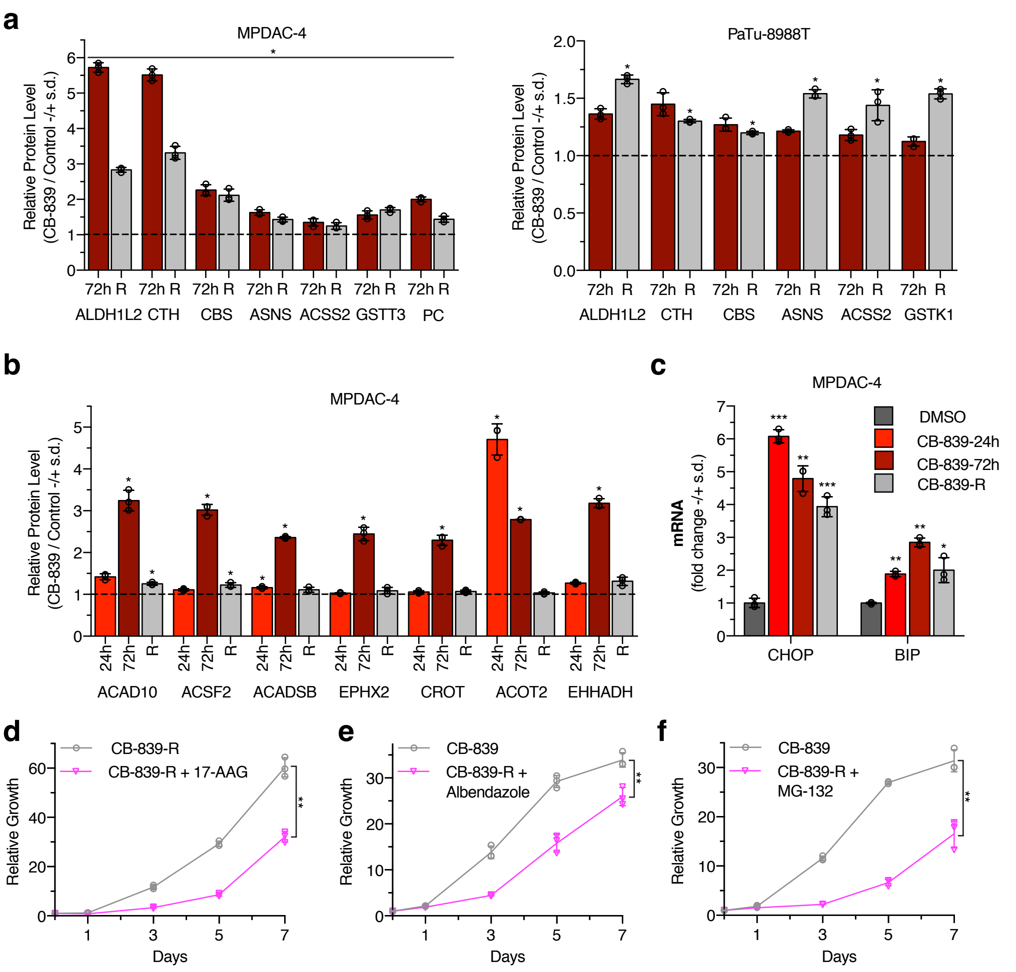


**Supplementary Figure 6 PDAC GLSi proteomics identifies altered metabolic and resistance pathways**
(**a**) Select oxidative stress response proteins and pyruvate carboxylase (PC) remain elevated in CB-839 resistant cells (minimum of 15 days of treatment). Data are derived from experimental data in Supplementary Data 6 and 9. Mean fold-change ± s.d. presented, for MPDAC-4 CB-839-72h and CB-839-R (Resistant) comparisons to DMSO were significant (* = FDR<0.05), for PaTu-8988T, all CB-839-R (Resistant) comparisons to DMSO were significant (* = FDR<0.05), P values in Supplementary Data 6 and 9 and tabulated for ease of viewing in Supplementary Data 7. (**b**) Fatty acid oxidation related enzyme levels in MDPAC-4 cells in response to CB-839, levels are elevated at 72 h but decrease close to baseline in CB-839-R cells. Data are derived from experimental data in Supplementary Data 4 (Experiment 1) and 9. Mean fold-change ± s.d. presented, all P values are tabulated in Supplementary Data 4 and 9 and for ease of viewing in Supplementary Data 7. All comparisons are made to DMSO values (* = FDR<0.05). (**c**) Markers of endoplasmic reticulum stress response assessed by RT-qPCR in MPDAC-4 cells treated with DMSO or CB-839 for the indicated times. Expression levels are normalized to 18S ribosomal RNA and presented as mean ± s.d. of 3 independent wells from a representative experiment (of 3 experiments). CHOP: CB-839-24h vs. DMSO, P=0.00001, CB-839-72h vs. DMSO, P=0.00136, CB-839-R vs. DMSO, P=0.00081. BiP: CB-839-24h vs. DMSO, P=0.0011, CB-839-72h vs. DMSO, P=0.00105, CB-839-R vs. DMSO, P=0.04402. Significance determined by t-Test, * P<0.05, ** P<0.01, *** P<0.001, ns: non-significant, P>0.05. (**d**) Relative proliferation of CB-839-R MPDAC-4 cell line treated with CB-839 with or without 17-AAG (500 nm), CB-839-R vs. CB-839-R + 17-AAG, Day 7, P=0.00143. (**e**) Relative proliferation of CB-839-R MPDAC-4 cell line treated with CB-839 with or without albenazole (Prestwick-675) (400 nm), CB-839-R vs. CB-839-R + albendazole, Day 7, P=0.00618. (**f**) Relative proliferation of CB-839-R MPDAC-4 cell line treated with CB-839 or DMSO with or without MG-132 (500 nm), CB-839-R vs. CB-839-R + MG-132, Day 7, P=0.00260. Data for d,e,f are plotted as mean relative cell proliferation, error bars depict ± s.d. of 3 independent wells from a representative experiment (of 3 experiments). Significance determined by t-Test in panels d,e,f comparing last time points. * P<0.05, ** P<0.01, ns: non-significant, P>0.05.

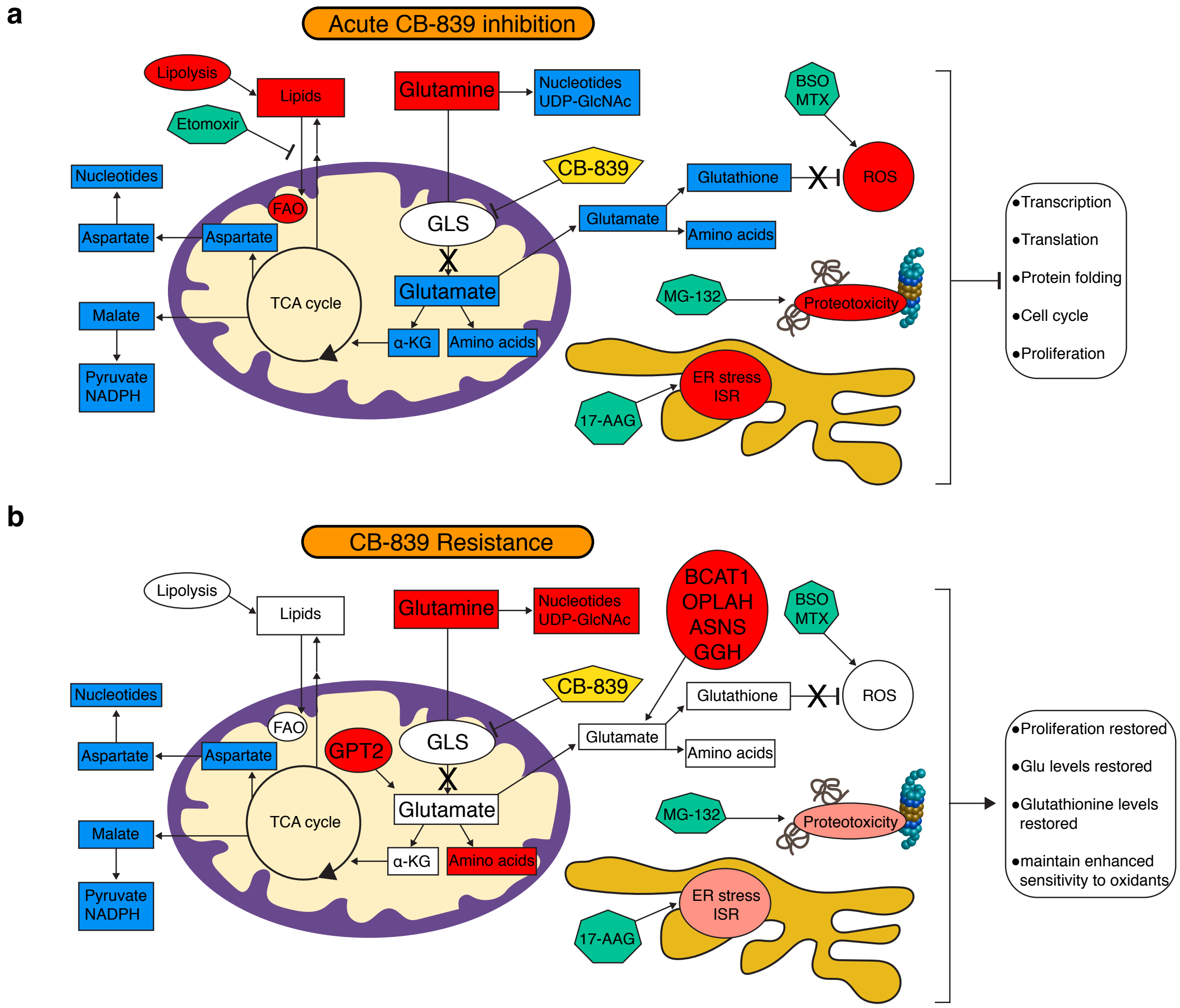


**Supplementary Figure 7 Model for effects of acute CB-839 inhibition and CB-839 resistance mechanisms**
(**a**) Acute CB-839 inhibition (4-72 h) leads to an initial significant decrease in glutamine derived metabolic pathways resulting in an increase in oxidative stress, proteotoxicity, and an integrated stress response/endoplasmic reticulum (ER) stress response. This precipitates a significant cellular response including importantly a marked decrease in proliferation. Blue = downregulated metabolites or processes, Red = upregulated metabolites or processes, White = unchanged. This schematic represents a composite of data derived from this study including metabolomics, proteomics, GSEA, and Connectivity Map 2.0 (CMAP) analysis. (**b**) Prolonged exposure to CB-839 leads to resistance pathways that allow PDAC cells to reestablish proliferation. Notably, multiple glutamate-producing enzymes (BCAT1, OPLAH, ASNS, GGH, and GPT2) likely contribute to a reaccumulation of baseline levels of Glutamate (Glu) in MPDAC-4 cells. While glutathione and reactive oxygen levels are restored to baseline levels, CB-839 resistant cells remain sensitive to oxidant-inducing drugs (BSO, MTX). According to CMAP analysis, RT-qPCR, and 17-AAG inhibitor results, the ER stress response is less active in CB-839-R cells (indicated by a lighter shade of red). Likewise, given proteasomal inhibition is less effective in CB-839-R cells, this is also indicated with a lighter shade of red. CB-839-R cells remain sensitive to proteasome inhibition. Abbreviations: FAO: Fatty acid oxidation, TCA Cycle: Tricarboxylic acid cycle, GLS: Glutaminase, α-KG: α-ketoglutarate, UDP-GlcNAc: Uridine diphosphate N-acetylglucosamine, ER stress (Endoplasmic reticulum stress), ISR: Integrated stress response, BSO: L-Buthionine-(S,R)-sulfoximine, MTX: Methotrexate, ROS: Reactive oxygen species, GPT2: alanine aminotransferase 2, BCAT1, branched chain aminotransferase 1 OPLAH: 5-oxoprolinase ASNS: asparagine synthetase, GGH: Gamma-Glutamyl hydrolase.

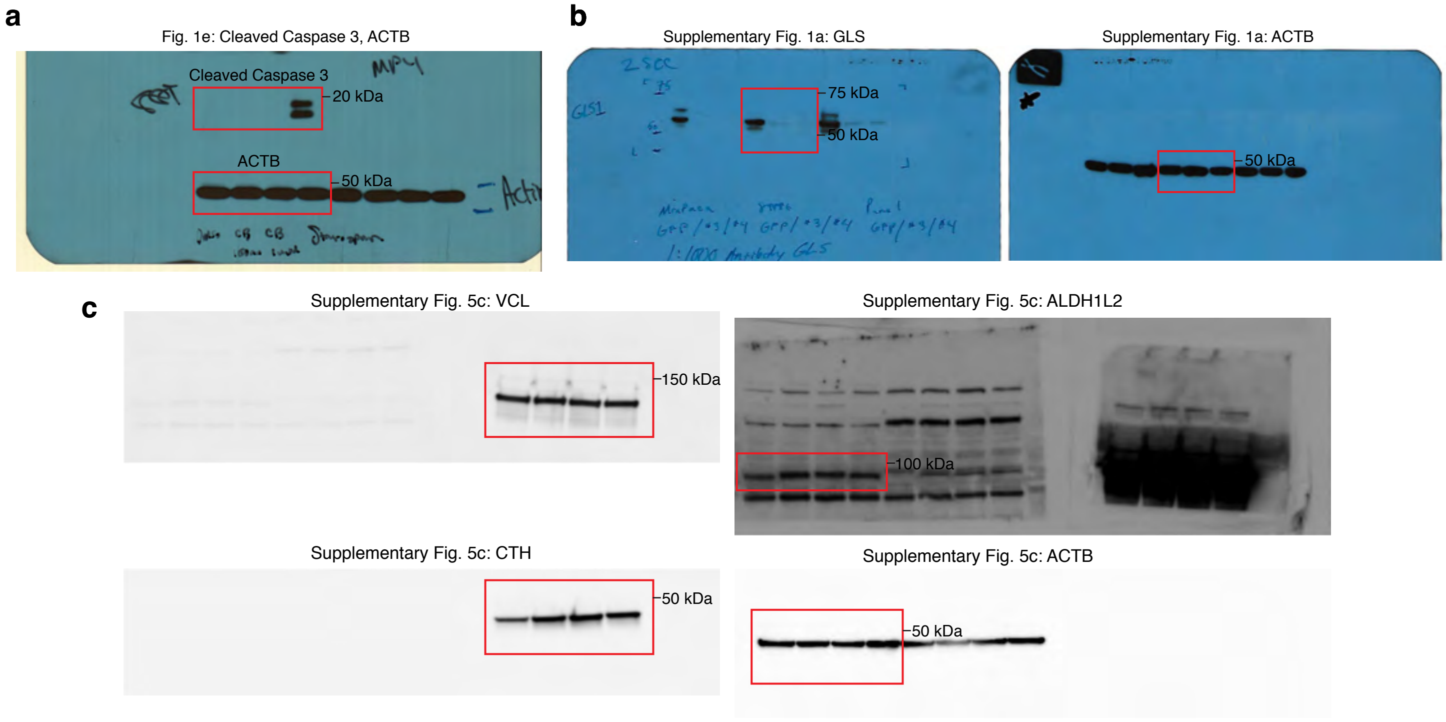


**Supplementary Figure 8: Uncropped immunoblots for all data elements shown in the manuscript**
(**a**) Uncropped immunoblots for Fig. 1e: Cleaved Caspase 3, ACTB. (**b**) Uncropped immunoblots for Supplementary Fig. 1a: GLS, ACTB. (**c**) Uncropped immunoblots for Supplementary Fig. 5c: VCL, ALDH1L2, CTH, ACTB.
